# PPE17 (Rv1168c) protein of *Mycobacterium tuberculosis* detects individuals with latent TB infection

**DOI:** 10.1371/journal.pone.0207787

**Published:** 2018-11-26

**Authors:** Philip Raj Abraham, Kamakshi Prudhula Devalraju, Vishwanath Jha, Vijaya Lakshmi Valluri, Sangita Mukhopadhyay

**Affiliations:** 1 Laboratory of Molecular Cell Biology, Centre for DNA Fingerprinting and Diagnostics (CDFD), Hyderabad, India; 2 Division of Immunology and Molecular Biology, LEPRA Society-Blue Peter Public Health and Research Center, Hyderabad, India; 3 Graduate Studies, Manipal Academy of Higher Education, Manipal, Karnataka, India; Universita Cattolica del Sacro Cuore, ITALY

## Abstract

Latent tuberculosis infection (LTBI) is a clinically distinct category of *Mycobacterium tuberculosis* (Mtb) infection that needs to be diagnosed at the initial stage. We have reported earlier that one of the Mtb proline-proline-glutamic acid (PPE) proteins, PPE17 (Rv1168c) is associated with stronger B-cell and T-cell responses and could be used to diagnose different clinical categories of active TB patients with higher specificity and sensitivity than PPD and ESAT-6. Based on these observations we further tested the potential of PPE17 for the diagnosis of LTBI. We tested 198 sera samples collected from LTBI individuals (n = 61), QFT-negative (n = 58) and active TB patients (n = 79). Individuals were defined as LTBI by QuantiFERON-TB Gold In-Tube test (QFT–GIT) positive results, while active TB patients were confirmed based on the guidelines of the Revised National TB Control Programme of India. The antibody responses against PPE17, ESAT-6:CFP-10 and PPD were compared in these subjects by enzyme-linked immunosorbent assay. We observed that LTBI individuals show a higher sero-reactivity to PPE17 as compared to currently used latent TB diagnostic antigens like ESAT-6, CFP-10 and PPD. The LTBI and active TB patients display almost similar sensitivity. Interestingly, PPE17 could discriminate LTBI positive subjects from the QFT-negative subjects (*P* < 0.001). Our study hints that PPE17 may be used as a novel serodiagnostic marker to screen the latently infected subjects and may also be used as a complimentary tool to the QFT–GIT.

## Introduction

Tuberculosis (TB) remains a global health problem with an estimated 10.4 million new cases of *Mycobacterium tuberculosis* infection and 1.7 million deaths during 2016 [1]. Diagnosis of TB is made by simple technique like sputum smear microscopy to highly advanced molecular test such as GeneXpert MTB/RIF assay where infection with Mtb and its resistance to first-line anti-TB drug, rifampicin are detected simultaneously. However, diagnostic challenges arise in case of asymptomatic Mtb infection such as latent TB infection (LTBI). Subjects with LTBI are sputum negative by definition. World Health Organization defines LTBI as a state of persistent immune response to Mtb antigens without evidence of clinically manifested active TB [2]. According to WHO, about one third of the global population is suffering from LTBI and there is a risk for these subjects towards progression of active form of TB. Further enormous reservoir of LTBI will be a major problem for global TB control. Therefore, improvements in the early diagnosis and treatment of LTBI are urgently needed to challenge this latent reservoir [3]. However, research gap exists in the development of diagnostic tests with improved performance and a gold standard for the diagnosis of LTBI is not available until now [4].

Diagnosis of LTBI is based on tuberculin skin test (TST) or blood test such as interferon-gamma release assays (IGRA), information gathered from the medical history, chest radiograph, physical examination, and examination of sputum in certain circumstances [5]. TST and IGRA measures host immunity to Mtb that represents an indirect “immunologic foot print” of past infection. In addition, these tests do not predict persons at higher risk of progression towards TB. Although, TST was used as a standard for screening latent tuberculosis infection until the early 2000s, limitations have been observed in terms of precise intradermal administration, follow-up visit to interpret the test results and also the possibility of false-positive results with *M*. *bovis* Bacillus Calmette-Guerin (BCG) vaccination or other environmental mycobacteria [6]. At present, LTBI is mainly diagnosed by U.S. Food and Drug Administration (FDA) approved commercially available IGRA tests such as QuantiFERON–TB Gold In-Tube test (QFT–GIT) and SPOT TB test (T–Spot). These tests measure the levels of interferon-gamma (IFN-γ) released by cells of whole blood or number of cells producing IFN-γ, after *in vitro* stimulation with Mtb-specific antigens, early secreted antigenic target 6 kDa (ESAT-6; Rv3875), and culture filtrate protein 10 kDa (CFP-10; Rv3874) [7]. Since ESAT-6 and CFP-10, encoded by the region of difference 1 (RD1) are absent in BCG and most non-tuberculous mycobacteria, IGRA test appears to be specific without revealing any cross-reactivity unlike TST and therefore can be implemented to detect latent Mtb infection even in BCG-vaccinated healthy subjects [8, 9]. Nevertheless, limitations are also observed in these tests such as processing of blood samples within 8–30 hours of collection, decrease in accuracy of IGRA due to errors in collecting or transporting blood specimens and assay interpretation. Further, insufficient data are available on the use of IGRA for children younger than 5 years of age, persons recently exposed to Mtb and immunocompromised persons [5, 10]. Moreover, these tests are expensive and cannot be implemented in resource poor settings.

Proteins of Mtb are found to induce antibody responses in 90% of patients exposed to TB bacilli [11]. Due to simplicity and convenient detection of pathogens, low costs, easy operation and rapid determination, serological assays that measures antibody responses to selective Mtb antigens in suspected cases of TB are still considered to be attractive tools especially in resource limiting country like India [12, 13]. In this direction, efforts were made globally to identify potential serodiagnostic antigens that induce strong antibody responses in TB patients. Among these, proline-glutamic acid (PE)/proline-prolineglutamic acid (PPE) proteins of Mtb have been well studied as these proteins act as a source of antigenic variation. Deciphering the biology of Mtb genome revealed that approximately 10% of the coding capacity of the Mtb genome encodes proteins carrying PE or PPE motifs near the N-terminus region [14]. Several studies have indicated that the PPE proteins induce strong B-cell responses and could be used to diagnose active TB cases [15–19]. Earlier, we have demonstrated that the PPE17 (Rv1168c) protein of Mtb induce strong B-cell and T-cell responses in active TB patients [17, 20, 21]. Interestingly, PPE17 was found to display stronger immunoreactivities against sera obtained from clinically active TB patients compared to PPD, Hsp60 and ESAT-6 proteins and could distinguish TB patients from the BCG-vaccinated healthy controls [17]. PPE17 is shown to overexpress during Mtb infection [22]. Also, we observed that sera from active TB patients showed higher reactivity against PPE17 compared to other PPE protein like PPE2 [20] and antibody responses in patients with active TB are mostly directed towards the N-terminal domain of PPE17 [21]. We demonstrated earlier that antibodies directed against the N-terminal domain of PPE17 in active TB patients did not cross-react with N-terminal domains of other PPE proteins [21]. However, the potential role of PPE17 in discriminating subjects with latent TB infection has not been studied earlier. Therefore, in the present study we have tested whether higher antibody responses are elicited against PPE17 in LTBI subjects also and whether PPE17 can be used as a serodiagnostic marker to discriminate LTBI positives cases from the QFT-negative subjects.

## Materials and methods

### Ethics statement

The study was approved by the Institutional Ethics Committee of LEPRA Society, Hyderabad. Before collecting the specimen, informed consent was obtained from the study participants.

### Collection of samples

A total of 198 samples were included in this study. These samples comprised of sera from individuals diagnosed as LTBI QFT-positive (n = 61), QFT-negative (n = 58) and active TB patients (n = 79). LTBI individuals includes 31 male and 30 female participants (mean age 32.79 years) while, QFT-negative subjects includes 30 male and 28 female participants (mean age 30.85 years). These LTBI individuals were diagnosed by QFT-GIT at the Blue Peter Public Health and Research Centre, Hyderabad, India. Furthermore, they were clinically categorized without bacteriological evidence and were confirmed to be positive by the QFT-GIT. Sera from active tuberculosis patients were collected from the DOTS (directly observed treatment short course) centre of Mahavir Hospital and Research Centre, Hyderabad, India. The guidelines of the Revised National TB Control Programme of India were followed for diagnosis of these patients (http://www.tbcindia.org) as described earlier [21].

### Purification of recombinant PPE17 protein

Cloning, expression and purification of recombinant PPE17 and its N-terminal (N-PPE17) protein were carried out following the method described by us earlier [17, 21]. Briefly, an open reading frame corresponding to PPE17 or N-PPE17 was cloned in pRSET-A vector (Invitrogen, Carlsbad, CA) and transformed in *Escherichia coli* BL21 (DE3) expression system. Polyhistidine-tagged recombinant PPE17 and N-PPE17 proteins were purified using TALON metal affinity resin (Takara Bio Inc. CA, USA). Similarly, ESAT-6:CFP-10 complex also purified as previously described by us [23]. After estimating the concentrations of these recombinant proteins, the preparations were aliquoted in microcentrifuge tubes and used within a week for assessing the antibody responses in sera samples.

### Enzyme-linked immunosorbent assay (ELISA)

Antibody response to recombinant PPE17 protein was measured in various sera samples following ELISA as described by us earlier [17, 20]. Briefly, PPE17 protein (1 μg/well; diluted in 0.1 M carbonate buffer of pH 9.5) immobilized 96-well microtiter plates (Costar, Corning, NY, USA) were incubated overnight at 4°C. Next day, after washing the plates with phosphate buffered saline (PBS, pH 7.4), the wells were blocked with 200 μl PBS containing 2% bovine serum albumin (blocking buffer) and kept for incubation at 37°C for 2 hours. After washing the ELISA plates for 3 times with PBS-Tween buffer (PBS-T), 50 μl sera samples were added and the plates were further incubated for 1 hour at 37°C. To detect the PPE17-specific antibodies, 50 μl of anti-human immunoglobulin G (IgG) conjugated horseradish peroxidase (HRP) was added (1/8000 dilution) and the plates were incubated for 1 hour at 37°C. In the next step, the anti-human IgG-HRP activity was measured by *o*-phenylenediamine tetrahydrochloride. Finally, the reaction was terminated with 1N H_2_SO_4_ and absorbance values were measured at 492 nm with an ELISA reader (BioTek Instruments Inc., VT, USA). Inhibition ELISA was carried out using 500 nM recombinantly purified N-terminal PPE17 as described by us earlier [21].

### Statistical analysis

For calculating the sensitivity of the assay, the mean ELISA absorbance values (OD at 492 nm (OD_492_)) of the QFT-negative subjects’ sera plus three or five standard deviation was calculated to determine the cut-off values. A sample is considered positive if it is showing antibody levels greater than or equal to the cut-off value. Statistical significance (*P* value) was calculated by non-parametric Mann Whitney test using GraphPad prism and *P* < 0.05 was considered as significant.

## Results

### PPE17 strongly discriminates LTBI individuals from QFT-negative subjects

**Fig 1:**
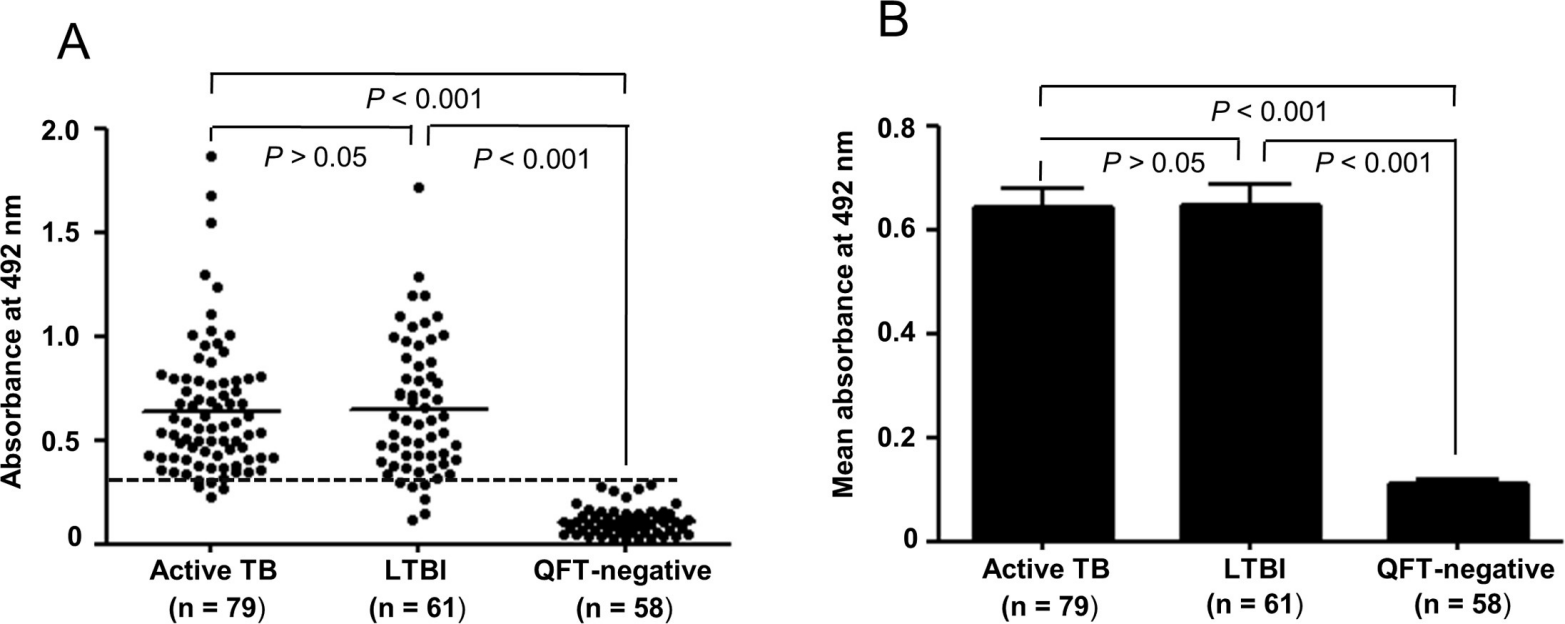
PPE17 (Rv1168c) displays higher antibody responses in individuals with active TB and latent TB. (A). Mean absorbance values (indicated by horizontal line) of PPE17 for active TB and LTBI individuals as well as QFT-negative subjects were compared following ELISA. (B). Data shown for individual samples in Fig 1A were re-plotted to show the mean ± SEM values. The mean ± SEM values for active TB, LTBI and QFT-negative subjects were 0.646 ± 0.035, 0.648 ± 0.041 and 0.113 ± 0.008 respectively.

In our previous study, we observed that PPE17 displayed immunoreactivities to active TB patients and could discriminate patients with active TB from BCG-vaccinated healthy control [20]. However, it is not clear whether PPE17 can also screen individuals with latent TB infection. Therefore, the levels of PPE17-specific antibodies were estimated in the sera collected from LTBI positive and compared with that of sera collected from QFT-negative subjects. On comparing the antibody responses to PPE17 among LTBI positive and QFT-negative subjects as well as active TB patients we observed that samples from active TB and LTBI positive groups induced higher antibody responses against PPE17. Albeit, TB and LTBI are clinically distinct categories of Mtb infection, there is no significant difference observed in the mean absorbance values between these two categories (mean OD_492_ ± SEM, 0.646 ± 0.035 *versus* 0.648 ± 0.041; *P >*0.05) (Fig 1A and 1B). Interestingly, QFT-negative subjects displayed poor antibody responses to PPE17 when compared with LTBI subjects (mean OD_492_ ± SEM, 0.113 ± 0.008 *versus* 0.648 ± 0.041; *P <*0.0001) (Fig 1A and 1B). These results suggest that PPE17 can elicit stronger antibody responses similar to active TB patients and can discriminate subjects with latent TB infection from the QFT-negative subjects.

**Fig 2:**
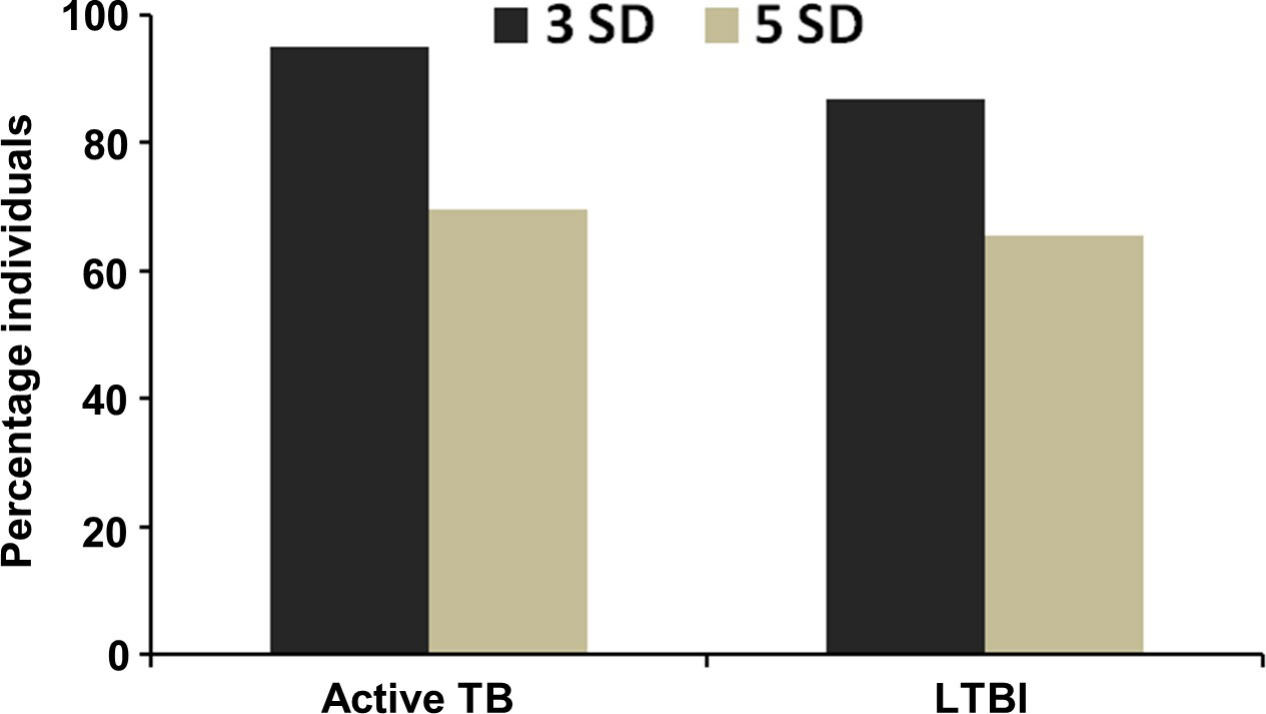
PPE17 is more sensitive to detect active TB and LTBI individuals. The percentages of high-level responders (samples showing antibody levels greater than or equal to the cut off values) shown in Fig 1A were calculated using a standard cut off value (mean OD_492_ of the QFT-negative sera plus 3 SD or 5 SD) for active TB and LTBI individuals.

Further, to test the sensitivity of PPE17 for diagnosing the LTBI individuals, a standard cut-off value was calculated using the mean OD_492_ of LTBI negative sera (mean OD_492_ (SD) value used for calculation of cut-off was 0.113 (0.065)) plus 3 SD and we observed that PPE17 could detect 86.88% (53/61) of LTBI individuals. Next, we have used a more stringent cut-off value (patients showing antibody levels greater than or equal to mean OD_492_ of LTBI negative sera plus 5 SD) to test the sensitivity of PPE17 for detecting LTBI individuals. When the proportion of high-level responders was analyzed, PPE17 could detect 65.57% (40/61) of individuals with latent TB infection (Fig 2). These values are almost similar to the values obtained in case of active TB infection (94.93% (75/79) in case of 3 SD and 69.62% (55/79) in case of 5 SD) (Fig 2). These results indicate that LTBI individuals mounted high antibody responses against PPE17 and like active TB patients, PPE17 is also sensitive for the diagnosis of latently infected TB individuals.

### PPE17 protein induces stronger antibody responses in LTBI as compared to ESAT-6:CFP-10 and PPD

**Fig 3:**
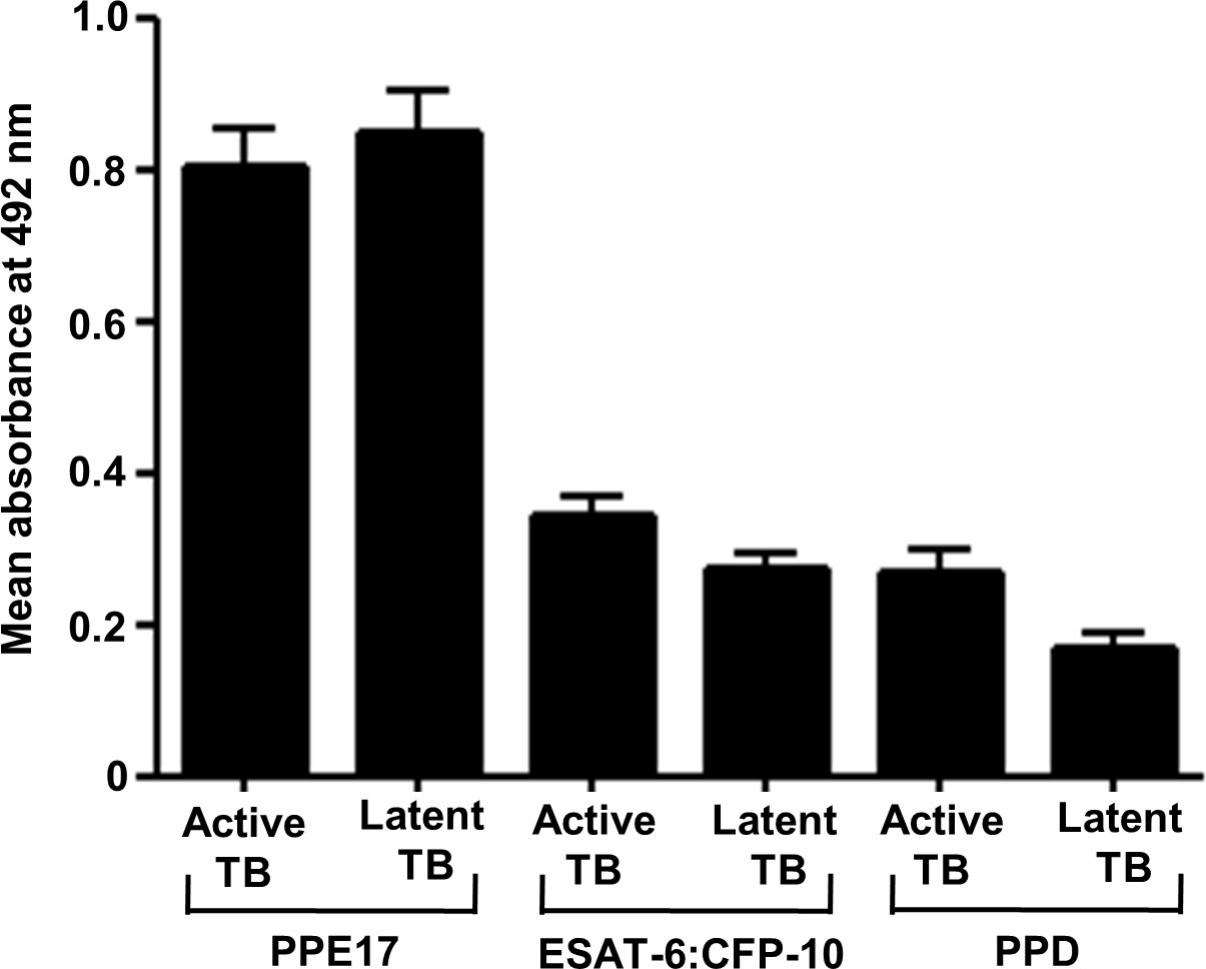
PPE17 elicits stronger antibody responses in active TB and LTBI compared to ESAT-6: CFP-10 and PPD. (A). Antibody responses to *M*. *tuberculosis* antigens PPE17, ESAT6:CFP10 and PPD were compared for LTBI individuals and active TB patients following ELISA. Results shown are in mean ± SEM.

As we observed that PPE17 displayed higher antibody responses in active TB and LTBI (Figs 1 and 2), it was of interest to compare the immunoreactivity of LTBI sera against PPE17 *versus* other Mtb antigens such as ESAT-6:CFP-10 and purified protein derivative (PPD) that are used in IGRA and TST for diagnosis of LTBI [5]. Interestingly, PPE17 displayed a higher mean absorbance value against LTBI samples as compared to ESAT-6:CFP-10 and PPD (mean OD_492_ ± SEM; 0.852 ± 0.054, 0.275 ± 0.018 and 0.170 ± 0.021 for LTBI) like active TB cases (mean OD_492_ ± SEM; 0.803 ± 0.052, 0.344 ± 0.027 and 0.277 ± 0.026) (Fig 3). Further, when we re-analyzed this data to test the immunoreactivities of individual sample against PPE17, ESAT-6:CFP-10 and PPD, it was interesting to note that majority of the active TB and LTBI samples displayed higher antibody responses to PPE17.

### The N-terminal domain of PPE17 (N-PPE17) protein induce antibody responses similar to the full-length PPE17 protein in LTBI individuals

**Fig 4:**
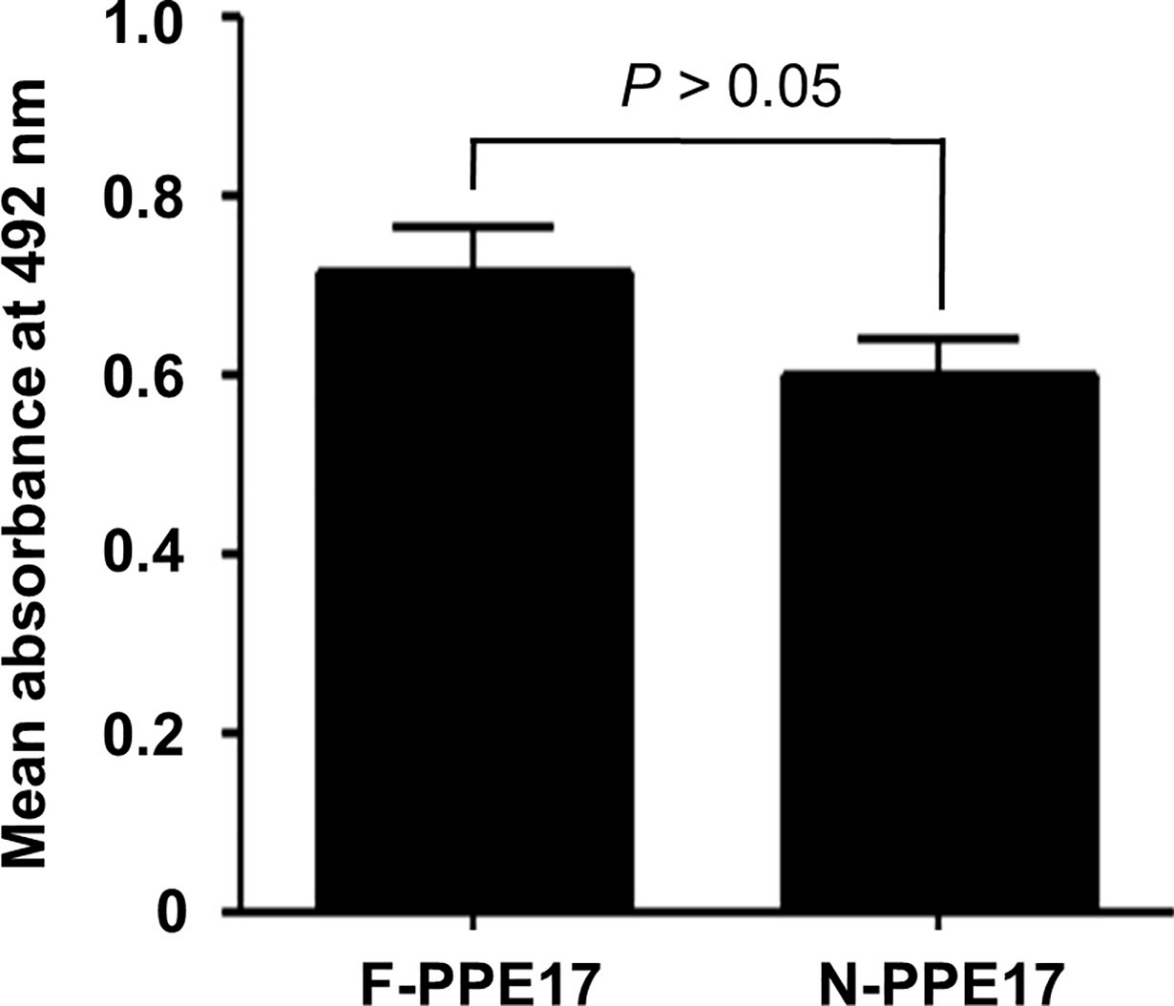
Both full-length PPE17 (F-PPE17) and N-terminal domain of PPE17 (N-PPE17) proteins display higher antibody titers in LTBI individuals. The mean absorbance values (OD_492_) of LTBI individuals were compared for F-PPE17 and N-PPE17 proteins following ELISA.

**Fig 5:**
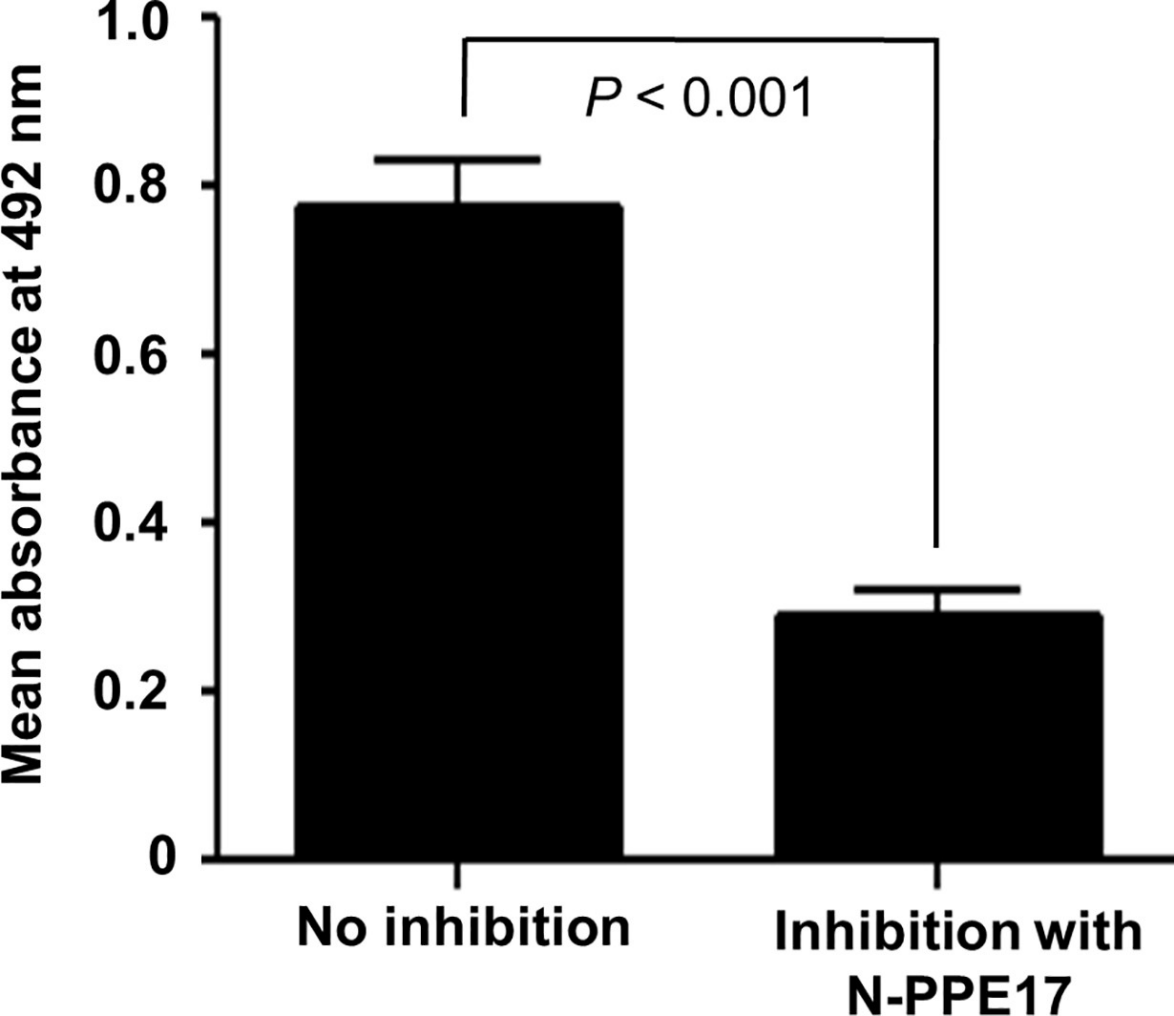
The N-terminal fragment of PPE17 (N-PPE17) predominantly recognize LTBI individuals. (A). The sera of LTBI individuals pre-incubated with 500 nM of N-PPE17 protein were added to ELISA plates coated with 500 nM of F-PPE17 protein. After washing, the plates were incubated with anti-human IgG-HRP. Antibody response was measured as a function of absorbance read at 492 nm (OD_492_) using chromogenic substrate OPD and H_2_O_2_ as substrate.

In our earlier studies, we found that the N-terminal domain of PPE17 (N-PPE17) actually plays a dominant role in inducing antibody responses in active TB patients [21]. To check whether antibody responses elicited in LTBI individuals are directed predominantly against the N-terminal domain of PPE17, we compared the antibody responses mounted against full-length PPE17 versus N-terminal domain of PPE17 (N-PPE17). Once again, we observed that in LTBI individuals, the N-PPE17 displayed almost similar antibody responses as observed in case of full-length PPE17 protein (mean OD_492_ ± SEM, 0.715 ± 0.051 for PPE17 and 0.601 ± 0.041 for N-PPE17 respectively; *P* > 0.05) (Fig 4). To further confirm the fact that antibodies to PPE17 in LTBI individuals are predominantly directed against the N-terminal domain of PPE17, we next carried out an inhibition ELISA where the LTBI sera were pre-incubated with 500 nM of recombinantly purified N-PPE17 before being added to the ELISA plates coated with 500 nM of recombinantly purified full-length PPE17 protein. As expected, N-PPE17 inhibited antibody responses to full-length PPE17 protein by about 75%. (mean OD_492_ ± SEM, 0.778 ± 0.053 for no inhibition *versus* 0.288 ± 0.029 for inhibition with N-PPE17; *P <* 0.001; Fig 5).

## Discussion

Latent TB infection is caused by *M*. *tuberculosis* in a state of non-replicating persistence. At present, LTBI diagnosis relies mainly on two tests, the century-old tuberculin skin test (TST) and US FDA approved, commercial IGRA test. The detection of LTBI cases based on the TST is less sensitive as well as non-specific while, limitations associated with IGRA based tests necessitates additional screening test for routine diagnosis of LTBI especially in resource limited settings. In contrary, rapid diagnostic tests such as serological assays are considered to be simple and inexpensive methods of diagnosis. It is true that the serological test, especially in case of TB is very useful for rapid diagnosis, prognosis and also to follow-up patients [13, 24]. Therefore, antibody-based diagnostic tests may be explored as an additional tool to screen LTBI subjects. In this direction, our study provides hope that PPE17 may be used as a novel serodiagnostic marker to screen the latently infected subjects.

We screened the sera samples collected from LTBI positive and QFT- negative subjects (based on QFT-GIT assay) for the presence of anti-PPE17 antibody. Interestngly, we observed that QFT-GIT positive subjects (LTBI positive) had higher anti-PPE17 antibody as compared to QFT- negative subjects (LTBI negative). It is pertinent to mention that in Mtb, PPE17 exists as a single gene; although it is present in *M*. *bovis*, a frame shift due to a single base insertion splits it into two parts. Thus, very low or no false positivity is expected in healthy control group as compared to subjects infected with Mtb. Interestingly, we observed that subjects with latent TB infection had almost similar mean antibody responses as observed in active TB patients. This indicated that probably PPE17 is expressed during latency. In fact, PPE17 is reported to be a surface exposed protein [25, 26] and is shown to be induced during non-replicating persistence as well as in conditions that mimic macrophage environment [22, 27]. Since LTBI is diagnosed by either TST or IGRA test, we next compared serodiagnostic potential of PPE17 with that of ESAT-6:CFP-10 and PPD, and PPE17 was found to display higher antibody responses in LTBI subjects as compared to ESAT-6:CFP-10 and PPD. We have also observed that LTBI subjects had almost identical antibody responses against F-PPE17 and N-PPE17 and antibody responses to F-PPE17 were significantly inhibited by N-PPE17. This suggests that like active TB patients, in LTBI also a higher antibody response is induced against N-PPE17.

It is essential to mention that PPE17 may be considered as a potent serodiagnostic marker for primary screening of LTBI subjects in addition to QFT-GIT test since an increased antibody response is mounted against PPE17 in these subjects. In fact, the sensitivity was found to be about 87% which is almost similar to the sensitivity observed for active TB patients (about 95%). It is also important to note that about 13% (8/61) of LTBI subjects detected by QFT-GIT assay could not be diagnosed by PPE17. The probable reason for this observation could be the differences in genetic polymorphism in these subjects which results in differences in B and T-cell responses [28, 29]. However, our results highlights that 10.34% (6/58) of subjects tested negative by QFT-GIT was positive for PPE17. Consequently, it is necessary to follow-up these subjects (tested negative by QFT-GIT but positive for PPE17) who may progress to active TB in near future.

Since PE/PPE proteins of Mtb are known to be source of antigenic variation, various studies have been undertaken to investigate the diagnostic potential of these proteins to detect both active TB and LTBI subjects. Koh et al., [30] studied the seroreactivity to PE-PGRS17 and PE-PGRS62 proteins. They found that PE-PGRS62 is associated with latent and active TB and the epitopes which are responsible for elicitation of humoral responses are present in the PGRS but not in the PE domain. Another study regarding expression of Mtb PPE44 gene among clinical isolates showed a sensitivity of 35.5% in patients with active tuberculosis and 37.5% in healthy subjects with latent tuberculosis infection [31]. In contrary, PPE17 showed a higher sensitivity of about 87% for detection of LTBI subjects in our study. One of the highly immunogenic PPE protein, PPE55 (Rv3347c) was reported to be expressed during incipient and clinical TB and suggested to be useful for differentiating between latent TB and incipient, subclinical TB [32]. Another group studied IL-2 response against PE35 and PPE68 between active TB patients and LTBI and concluded that PPE68 induced IL-2 could be used as a sensitive and specific biomarker for discriminating TB from LTBI [33]. All these studies made an effort to identify PE/PPE proteins to be used as diagnostic antigen for LTBI, however, major limitations of these studies was the use of low number of samples from the LTBI group. Further, the sensitivity observed in these studies is relatively lower. In summary, our study highlights use of the full-length or the N-terminal domain of PPE17 as a serodiagnostic marker to discriminate subjects with latent TB infection from LTBI negative healthy subjects. Although PPE17 could not discriminate active TB cases from LTBI, it may be used as a candidate antigen for screening Mtb infected from the uninfected healthy subjects. In this direction, our study provides hope that PPE17 may be used as a novel serodiagnostic marker to screen the latently infected subjects.
